# Association between Nutritional Behaviours and Acne-Related Quality of Life in a Population of Polish Male Adolescents

**DOI:** 10.3390/nu14132677

**Published:** 2022-06-28

**Authors:** Katarzyna Łożyńska, Dominika Głąbska

**Affiliations:** Department of Dietetics, Institute of Human Nutrition Sciences, Warsaw University of Life Sciences (SGGW-WULS), 159C Nowoursynowska Street, 02-776 Warsaw, Poland; katarzyna_lozynska@sggw.edu.pl

**Keywords:** acne vulgaris, juvenile acne, dermatological diseases, severity of acne, diet, nutrition, adolescents, quality of life

## Abstract

Acne vulgaris is diagnosed in the majority of adolescents, decreasing their quality of life, while the diet may influence its aetiology in a gender-dependent manner. The aim of the study was to analyse associations between nutritional behaviours and acne-related quality of life in a population of Polish male adolescents. The study was conducted on a population of Polish secondary school adolescents (a studied sample of 925 adolescents), while the random quota sampling procedure of secondary schools was applied. To assess acne-related quality of life, the Acne Quality of Life (AQoL) Scale and Acne Disability Questionnaire (ADQ) were applied, while the Social Quality of Life (SOCQOL) Score and Cardiff Acne Disability Index (CADI) were calculated. To assess the diet, an Acne-specific Food Frequency Questionnaire (Acne-FFQ) was applied. Neither for the ADQ results, nor for the CADI calculated on the basis of ADQ, was there an association with dietary intake (*p* > 0.05). The results of the SOCQOL Score (calculated on the basis of AQoL) were positively correlated with the intake of fish (*p* = 0.0085; R = 0.1144), salty snacks (*p* = 0.0495; R = 0.0854), and non-chocolate confectionary (*p* = 0.0078; R = 0.1156). In a group of respondents declaring any acne-related quality of life problems in AQoL, while compared with those declaring no such problems, higher intakes of dairy beverages other than milk (*p* = 0.0063), white bread (*p* < 0.0001), other white cereal products (*p* < 0.0001), fast foods (*p* = 0.0006), salty snacks (*p* < 0.0001), chocolate confectionary (*p* < 0.0001), and other confectionary (*p* < 0.0001), but lower intake of wholegrain bread (*p* = 0.0084) were observed. It may be concluded that acne-related quality of life is associated with dietary intake in a population of Polish male adolescents. In the studied population, the most prominent influencing factors were salty snacks and non-chocolate confectionary, with both of them having a proacnegenic effect.

## 1. Introduction

Acne vulgaris is defined as a chronic inflammatory disorder of the pilosebaceous unit, being common in the period of adolescence and until the early thirties [1]. The highest prevalence of acne is reported in Western Europe, North America, and Latin America. Taking into account its global prevalence, it is indicated as the 8th most common disease in the world [2]. The highest frequency of acne is stated at the age of 15, both for female and male individuals, while the decline in prevalence is stated in late adolescence, being consistent with the age of puberty completion (15–17 years for female adolescents and 16–17 years for male adolescents) [3]. In Poland, acne is diagnosed in about 75% of adolescents, without any difference of frequency between female and male individuals, and it decreases the quality of life in both genders [4]. Taking this into account, the psychosocial impact of acne is significant, and it may influence emotions, everyday activities, social life, study, as well as interpersonal relationships of adolescents [5].

The aetiology of acne is indicated to be multifactorial, as increased sebum secretion, endocrinological factors (including androgens), abnormal keratinization of the follicular infundibulum, bacterial proliferation, and subsequent inflammation are considered [6]. The possible influence of diet is also taken into account, and it is reflected in a recently growing trend of interest in this subject [7]. The prominent results for various food product groups are indicated, including high glycemic index foods, dairy products, fatty foods, and chocolate as potential acne-promoting factors, as well as specific fatty acids, fruit, and vegetable intake as potential acne-preventing ones [8]. The results are explained by the association between the intake of specific food products and sebum overproduction, or skin deterioration caused by the presence of hormones (for acne-promoting factors), as well as a reduction in inflammation (for acne-preventing ones) [9].

The recent systematic review by Meixiong et al. [10] indicated that high glycemic index and carbohydrate intake may have a modest yet significant proacnegenic effect, while the influence may be dependent on gender and ethnicity, so further studies are needed. Similar conclusions were formulated within a Polish study by Morze et al. [11], as a Western diet was associated with a higher acne intensity, but within this study the quality of life was not assessed. Taking this into account, the aim of the study was to analyse the association between nutritional behaviours and acne-related quality of life in a population of Polish male adolescents.

## 2. Materials and Methods

### 2.1. Ethical Statement

The study was carried out at the Department of Dietetics, Institute of Human Nutrition Sciences, Warsaw University of Life Sciences (WULS–SGGW). It was conducted on a population-based sample of Polish male adolescents, according to the guidelines laid down in the Declaration of Helsinki. All procedures involving human subjects received the approval of the Ethics Committee of the Institute of Human Nutrition Sciences of the Warsaw University of Life Sciences (No. 23/2020). All of the participants, as well as their parents or legal guardians, provided informed consent to participate in the study.

As the highest frequency of acne for male individuals is stated at the age of 15, while in late adolescence the decline in prevalence is stated as being consistent with 16–17 years [3], the study was planned to be conducted among boys aged 13–20 years (being consistent with the age attributed to a period of a secondary school education in Poland).

### 2.2. Studied Population

The study was conducted on a population-based sample of Polish male adolescents recruited within the secondary schools, and the planned age of the studied group was 13–20 years. It results from the fact that in Poland the typical age in secondary schools is 14–19 years, but participants one year younger (13 years—possibility of the admission of children one year younger) and one year older (20 years—possibility of extended education) were intended to be included.

In Poland, the Net Enrolment Rate (NER) for secondary education is 89.38% [12], so the recruitment of a population-based sample of adolescents within schools is a commonly applied procedure [13]. To obtain a representative national Polish sample of adolescents, secondary schools from Poland were randomly selected while using random quota sampling, and principals were invited to have their school participate in the study. The recruitment of male adolescents to the study was carried out in the randomly selected schools, while participation was voluntary.

The secondary schools were selected within two-stage random selection—within each voivodeship (basic administrative unit; 16 voivodeships in Poland), five random counties were chosen (total number of 16 × 5 = 80 counties), and within each county, five random secondary schools were chosen (total number of 80 × 5 = 400 secondary schools). The applied procedure was developed in order to obtain equal representation of the schools from large cities and small towns. 

The inclusion criteria were as follows:

- Student of the randomly selected secondary school;
- Polish ethnicity;
- Male gender;
- Aged 13–20 years;
- Informed consent to participate provided by student and their parent/legal guardian.

The students meeting the inclusion criteria received a link to an electronic version of the questionnaire.

The exclusion criteria were as follows:

- Any diet-related disease which may have interrupted the habitual diet;
- Any missing answers within the provided questionnaire;
- Any unreliable answers within the provided questionnaire.

**Figure 1:**
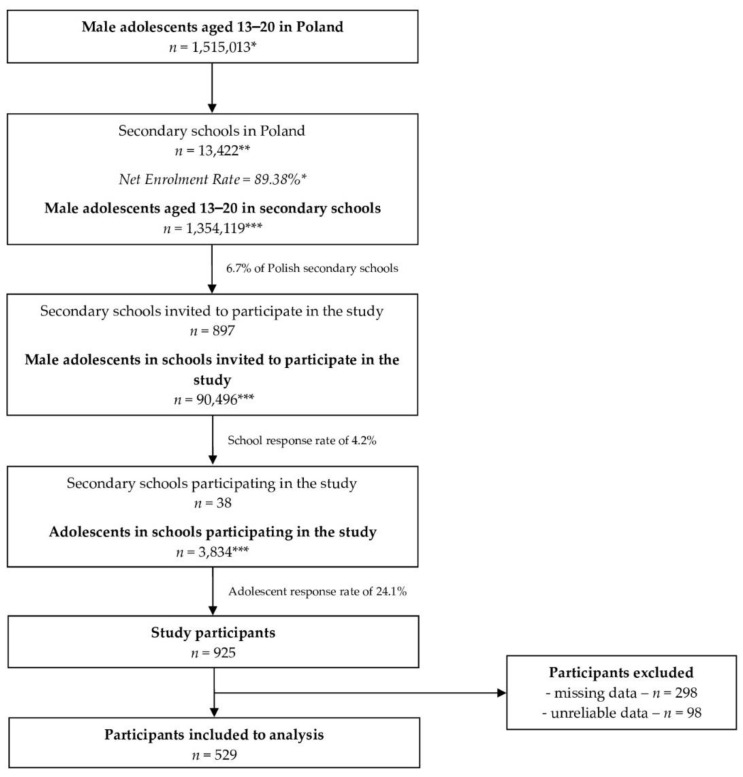
The detailed sampling and recruitment procedure; * data of the Central Statistical Office in Poland [12,14]; ** data of the Polish Ministry of National Education [15]; *** calculated based on available data.

The detailed sampling and recruitment procedure is presented in Figure 1. The final sample gathered within the study was 925 participants, while 529 of them were included in the analysis. The required minimum sample size was calculated as 384 based on the size of the population of male adolescents aged 13–20 years in Poland, of 1,515,013 for December 2020 (data of the Central Statistical Office in Poland [14]), for the assumed confidence level of 95% and the maximum error of 5%.

The studied population was recruited from all the regions of Poland from cities of various size, while the proportional distribution of participants was maintained, so it was considered representative.

### 2.3. Applied Questionnaire 

The study was conducted in the period of May 2021–February 2022, while using the Computer-Assisted Web Interview (CAWI) method and Google Forms tools. During the study, no sensitive or personal data were collected, and only the questions associated either with the aim of the study, or verification of the inclusion/exclusion criteria were asked. Based on the gathered data, identification of any participant of the study was impossible, so the questionnaire was considered anonymous. The time necessary to complete the study by a single respondent was estimated as 10 min, as it consisted mainly of close-ended questions, and some open-ended questions about frequency of consumption (food frequency questionnaire).

Within the study, the following questionnaires were used:

- The Acne Quality of Live (AQoL) Scale developed and validated by Gupta et al. [16];
- The Acne Disability Questionnaire (ADQ) developed by Motley et al. [17] and validated [18];
- The Acne-specific Food Frequency Questionnaire (Acne-FFQ), including food item questions about products being associated with acne course, as products playing role in either its development or prevention (vegetables, fruit, water, milk, other dairy beverages, white bread, wholegrain bread, other white cereal products, other wholegrain cereal products, fish, fast food, salty snacks, chocolate confectionary, other confectionary), included from the validated food frequency questionnaires: Ironic-FFQ [19], Iodine-FFQ [20], and Mg-FFQ [21];
- Basic questionnaire including questions about characteristics within inclusion/exclusion criteria.

Within the presented study, Polish versions of all questionnaires were applied, which were translated from English to Polish, with transcultural adaptation, if needed, according to the recommendations by the World Health Organization [22]. The process of translation included forward translation into Polish (conducted by the native Polish researcher, being fluent in English and familiar with the studied area), backward translation into English (conducted by the other researcher, not being familiar with the aim of the study in order to reduce bias), and an expert panel (including native Polish researchers, being fluent in English). The whole procedure was planned to improve the attainment of conceptual, semantic, idiomatic, and cultural equivalence.

Within the AQoL Scale, respondents were asked to specify the extent to which they experience the following feelings as a result of their acne: not feeling self-conscious in the presence of others; decrease in socialization with others; difficulties in relationship with partner; difficulties in relationship with close friends; difficulties in relationship with immediate family; feeling like an “outcast” most of the time because of the effect of acne upon appearance; people making fun of appearance; feeling rejected in romantic relationship because of the effect of acne upon appearance; and feeling rejected by friends because of the effect of acne upon appearance (9 items) [16]. Each of them was to be scored by respondent from 0 to 3 (0—not at all; 1—mildly; 2—moderately; 3—very markedly), while the Social Quality of Life (SOCQOL) Score was calculated as a mean value for 9 items [17].

Within ADQ, respondents were asked to specify the extent to which they experienced the following feelings as a result of their acne, and were asked about their opinions on their acne: feeling aggressive, frustrated, or embarrassed; feeling interfered with daily social life, social events, or relationships with members of the opposite sex; avoided public changing facilities or wearing swimming costumes; feelings about the appearance of skin; and how bad they think their acne is (5 items) [17]. For each of them, the answer was attributed to a score from 0 to 3, while the Cardiff Acne Disability Index (CADI) total score was calculated as a total value for 5 items [17].

Within the Acne-FFQ, respondents were asked to specify the exact number of servings of food items included that are consumed during a typical week (open-ended questions), while the serving sizes were described for each food item separately, as in the original food frequency questionnaires [19,20,21]. The number of servings was to be declared as a specific value (decimal parts were allowed).

### 2.4. Statistical Analysis

The distribution was verified using the Shapiro–Wilk test. Due to nonparametric distribution, the groups were compared using the Mann–Whitney *U* test, and the analysis of correlations was conducted using the Spearman rank correlation coefficient. The accepted level of significance was *p* ≤ 0.05. Statistica 13.0 (Statsoft Inc., Tulsa, OK, USA) was used for the statistical analysis.

## 3. Results

The baseline characteristics of the studied male adolescents are presented in Table 1. The majority of respondents were minors (76.18%), and lived in medium-sized cities (60.11%). Depending on the applied tool, a various share of respondents were attributed to any acne-related problems, as for the AQoL scale it was 37.80% for the social quality of life, and for the ADQ it was 75.61% for the general quality of life.

The results of the SOCQOL Scores obtained based on the AQoL scale in the studied group of male adolescent participants of the study are presented in Table 2. It was observed that for all assessed social quality of life items, the mean scores were rather low, but there were some respondents with high scores (a maximum value of 3 was obtained).

The results of the CADI obtained based on the ADQ in the studied group of male adolescent participants of the study are presented in Table 3. It was observed that for all assessed quality of life items, the mean scores were rather low, but there were some respondents with high scores (a maximum value of 3 was obtained).

The results of the Acne-FFQ in the studied group of male adolescent participants of the study are presented in Table 4. It was observed that a diverse population was gathered, characterised by various dietary patterns, as for each food item, there were respondents declaring either no consumption or very high consumption. 

The analysis of the correlation between intake of the acne-related food products obtained based on the Acne-FFQ and the results of the SOCQOL Score obtained based on the AQoL scale, as well as the CADI obtained based on the ADQ in the studied group of male adolescent participants of the study is presented in Table 5. It was observed that dietary intake was not associated with the results of the CADI (*p* > 0.05), but some associations were observed for the results of the SOCQOL Score. Positive correlations were observed for fish (*p* = 0.0085; R = 0.1144), salty snacks (*p* = 0.0495; R = 0.0854), and non-chocolate confectionary (*p* = 0.0078; R = 0.1156). At the same time, correlations close to statistical significance were observed for vegetables (*p* = 0.0734; R = −0.0779), white cereal products other than bread (*p* = 0.0842; R = 0.0752), wholegrain cereal products other than bread (*p* = 0.0801; R = 0.0762), and fast foods (*p* = 0.0646; R = 0.0804).

The analysis of the difference of dietary intake between male adolescent participants of the study declaring no acne-related quality of life problems and any acne-related quality of life problems within any question of the AQoL scale is presented in Table 6. It was observed that the dietary intake differed while compared between participants declaring no acne-related quality of life problems and any acne-related quality of life problems. The higher intake of dairy beverages other than milk (*p* = 0.0063), white bread (*p* < 0.0001), white cereal products other than bread (*p* < 0.0001), fast foods (*p* = 0.0006), salty snacks (*p* < 0.0001), chocolate confectionary (*p* < 0.0001), and other confectionary (*p* < 0.0001), but lower intake of wholegrain bread (*p* = 0.0084) were observed in a group of respondents declaring any acne-related quality of life problems, than for respondents declaring no acne-related quality of life problems.

The analysis of the difference of dietary intake between male adolescent participants of the study declaring no acne-related quality of life problems and any acne-related quality of life problems within any question of the ADQ is presented in Table 7. It was observed that dietary intake did not differ when compared with participants declaring no acne-related quality of life problems and any acne-related quality of life problems. 

## 4. Discussion

In the studied group of Polish male adolescents, the significant differences were stated, while compared associations between dietary intake and quality of life measured using various tools (ADQ and AQoL). Neither for the ADQ results, nor for the CADI calculated on the basis of ADQ, was there an association with dietary intake, while both for AQoL and SOCQOL Score calculated on the basis of AQoL, there were such statistically significant associations. This observation corresponds with the various shares of respondents declaring any acne-related quality of life problems for the studied questionnaires—for AQoL they were declared by 38.80% of respondents, while for ADQ they were declared by 75.61% of respondents. Even though both tools assess the social and emotional impacts of acne, the AQoL scale is less sensitive to any changes in self-declared indices of acne severity and the psychological morbidity that is associated with acne. Taking this into account, it may be indicated that in the studied group, the AQoL was a more suitable tool, as it allowed for the definition of a population of adolescents having more serious acne-related problems, while for the ADQ, for the majority of participants, such problems were indicated, so it was not a specific indicator. 

The ADQ as well as CADI calculated on the basis of the ADQ have been widely used in numerous studies with its psychometric properties verified, and in the recent systematic review by Smith et al. [23], it was identified to be among the top five most commonly used instruments for measuring the impacts of acne. However, in the review by Abdelrazik et al. [18], it was indicated that this questionnaire was so far verified mainly in the female populations, which was explained by the fact that women may be more likely to participate in such studies associated with acne. It should be indicated that the ADQ is based on five questions only, including those with the highest scorings in the studied group—about feeling aggressive, frustrated or embarrassed, feelings about the appearance of skin, and their opinion about how bad they think their acne is [17]. Simultaneously, AQoL consists of 9 or 12 questions (depending on the version), but it does not include questions about feeling aggressive, or frustrated due to acne, as well as about self-assessed acne [16], so it may explain the higher suitability of this tool in a studied population of male respondents. It may be explained by the fact that adolescent males may be, in general, more prone to aggressive reactions than female ones [24], as well as by the fact that adolescent males in older age groups are prone to experiencing psychological health symptoms caused by their body image [25].

In the studied population of male adolescents, there was observed an association between dietary intake of specific food groups and acne-related quality of life, while the negative influence of salty snacks and non-chocolate confectionary was stated in all the analyses conducted for AQoL. At the same time, for dairy beverages other than milk, fish, chocolate confectionary, white bread, other white cereal products, and fast foods, the negative effect, as well as for wholegrain bread—the positive effect, was indicated for some of the conducted analysis.

The proacnegenic effect of both salty snacks and non-chocolate confectionary is in agreement with the analysis of the other authors. The negative influence of salty snacks was also observed by the other authors, as they observed that the consumption of salty snacks is higher in individuals with acne than in those without [26]. Similarly, the consumption of products high in salt, namely chips [27], sausages, or burgers, was associated with an increased risk of acne [28]. It corresponds with the general opinion of adolescents, that salty products, such as chips, may aggravate their acne [29]. Such an association between salty products and acne development or progression may result from the fact that sodium is in general linked with a pro-inflammatory effect [30]. Moreover, the influence of sodium is explained by its role in the regulation of water and electrolyte homeostasis, including maintaining extracellular fluid volume [26]. In the case of a higher than recommended sodium intake, which may not be excreted and must be accumulated, such a situation leads to edema in the infundibulum of the hair follicles, and compression of the walls of the follicular ostea, accompanied by occlusion of the pilosebaceous orifice, being among the etiological mechanisms of acne [31].

Similarly, the negative influence of confectionary is confirmed by the studies of other authors. A lot of studies indicated that the intake of available carbohydrate and glycemic load of diets is higher in individuals with acne than in those without [32], as well as that low-glycemic-load diets may lead to alleviating acne symptoms [33]. It is confirmed by the results of studies for specific products, including sugary products and sugary beverages [34]. It corresponds with the general opinion of patients that food products high in sugar, such as chocolate, soda drinks, sugar, or all refined carbohydrates, may aggravate their acne [35]. This association is explained by the influence of refined carbohydrates on numerous factors related to acne development and progression, including insulin-like growth factor-1 (IGF-1) [36], IGF-binding protein, and androgen level [37]. Those factors, as a result, affect keratinocyte proliferation and apoptosis, as well as increase production of sebum [38].

In spite of the fact that the presented study revealed some valuable observations, it should be indicated that it was conducted during the COVID-19 pandemic, which may have influenced the observed results. It is well known that the COVID-19 pandemic influenced hand hygiene [39] and personal protective behaviours [40], including using face masks [41]. Taking this into account, it must be emphasised that the use of face masks, due to the COVID-19 pandemic, may trigger acne symptoms [42]. At the same time, it must be indicated that the pandemic period influenced the food habits of adolescents [43], including emotional eating behaviours [44], while both diet [10] and stress also influence acne [45]. However, as all indicated factors related to the COVID-19 pandemic were unified within the analysed group, it was assumed that their influence may be negligible within the studied population.

## 5. Conclusions

It may be concluded that acne-related quality of life is associated with dietary intake in a population of Polish male adolescents. In the studied population, the most prominent influencing factors were salty snacks and non-chocolate confectionary, both of which had a proacnegenic effect.

## Figures and Tables

**Table 1:** The baseline characteristics of the studied group of male adolescent participants of the study.

Variable		Male Responders (*n* = 529)
Age	Minors (age 13–17 years)	403 (76.18%)
	Adults (age 18–20 years)	126 (23.82%)
Size of city/town	Villages/small towns (<20,000 inhabitants)	126 (23.82%)
	Medium cities (20–100,000 inhabitants)	318 (60.11%)
	Big cities (>100,000 inhabitants)	85 (16.07%)
Acne Quality of Life (AQoL) scale	No acne-related quality of life problems	329 (62.20%)
	Any acne-related quality of life problems	200 (37.80%)
Acne Disability Questionnaire (ADQ)	No acne-related problems	129 (24.39%)
	Any acne-related problems	400 (75.61%)

**Table 2:** The results of the Social Quality of Life (SOCQOL) Score obtained based on the Acne Quality of Life (AQoL) scale in the studied group of male adolescent participants of the study.

Acne Quality of Life Scale		Mean ± SD	Median (Min–Max)
Acne Quality of Life scale items	Not feeling self-conscious in the presence of others	0.38 ± 0.72	0 * (0–3)
	Decrease in socialization with others	0.25 ± 0.62	0 * (0–3)
	Difficulties in relationship with spouse/partner	0.25 ± 0.66	0 * (0–3)
	Difficulties in relationship with close friends	0.18 ± 0.57	0 * (0–3)
	Difficulties in relationship with immediate family	0.18 ± 0.60	0 * (0–3)
	Feeling like an ‘outcast’ most of the time because of the effect of acne upon appearance	0.22 ± 0.61	0 * (0–3)
	People making fun of appearance	0.22 ± 0.62	0 * (0–3)
	Feeling rejected in romantic relationship because of the effect of acne upon appearance	0.24 ± 0.68	0 * (0–3)
	Feeling rejected by friends because of the effect of acne upon appearance	0.16 ± 0.57	0 * (0–3)
Social Quality of Life (SOCQOL) scale total score		0.23 ± 0.54	0 * (0–3)

* non-parametric distribution (Shapiro-Wilk test; *p* ≤ 0.05).

**Table 3:** The results of the Cardiff Acne Disability Index (CADI) obtained based on the Acne Disability Questionnaire (ADQ) in the studied group of male adolescent participants of the study.

Acne Disability Questionnaire (ADQ)		Mean ± SD	Median (Min–Max)
Acne Disability Questionnaire items	Feeling aggressive, frustrated or embarrassed	0.63 ± 0.84	0 * (0–3)
	Feeling interfered with daily social life, social events or relationships with members of the opposite sex	0.29 ± 0.70	0 * (0–3)
	Avoided public changing facilities or wearing swimming costumes	0.31 ± 0.77	0 * (0–3)
	Feelings about the appearance of skin	0.63 ± 0.78	0 * (0–3)
	How bad they think their acne is	0.82 ± 0.79	0 * (0–3)
Cardiff Acne Disability Index (CADI) total score		2.71 ± 2.95	2 * (0–15)

* non-parametric distribution (Shapiro–Wilk test; *p* ≤ 0.05).

**Table 4:** The results of the Acne-specific Food Frequency Questionnaire (Acne-FFQ) in the studied group of male adolescent participants of the study.

	Serving Size Described within the Food Frequency Questionnaire	Number of Servings per Week	
		Mean ± SD	Median (Min–Max)
Vegetables	80 g	6.42 ± 7.80	4 * (0–70)
Fruit	80 g	5.98 ± 6.93	4 * (0–45)
Water	250 g	25.46 ± 27.70	14 * (0–255)
Milk	250 g	4.09 ± 6.21	2 * (0–60)
Other dairy beverages	250 g	2.44 ± 2.88	2 * (0–24)
White bread	35 g	13.03 ± 13.55	8 * (0–70)
Wholegrain bread	35 g	4.68 ± 7.26	2 * (0–70)
Other white cereal products	70 g	4.20 ± 5.57	3 * (0–86)
Other wholegrain cereal products	70 g	2.29 ± 3.21	1 * (0–30)
Fish	100 g	1.18 ± 1.71	1 * (0–20)
Fast foods	1 meal	1.70 ± 2.39	1 * (0–35)
Salty snacks	1 serving	2.39 ± 2.30	2 * (0–14)
Chocolate confectionary	1 serving	2.69 ± 3.43	2 * (0–40)
Other confectionary	1 serving	2.55 ± 3.16	2 * (0–40)

* non-parametric distribution (Shapiro–Wilk test; *p* ≤ 0.05).

**Table 5:** Analysis of the correlation between the intake of acne-related food products obtained based on the Acne-specific Food Frequency Questionnaire (Acne-FFQ) and the results of the Social Quality of Life (SOCQOL) Score obtained based on the Acne Quality of Life (AQoL) scale, as well as the Cardiff Acne Disability Index (CADI) obtained based on the Acne Disability Questionnaire (ADQ) in the studied group of male adolescent participants of the study.

	Social Quality of Life (SOCQOL) Score		Cardiff Acne Disability Index (CADI)	
	*p*	R *	*p*	R *
Vegetables	0.0734	−0.0779	0.8572	−0.0078
Fruit	0.8188	0.0100	0.4253	0.0347
Water	0.9637	0.0020	0.9132	0.0047
Milk	0.4520	0.0328	0.6362	0.0206
Other dairy beverages	0.3424	0.0414	0.6349	0.0207
White bread	0.6808	0.0179	0.4949	−0.0297
Wholegrain bread	0.9413	0.0032	0.2705	0.0480
Other white cereal products	0.0842	0.0752	0.1351	0.0650
Other wholegrain cereal products	0.0801	0.0762	0.1539	0.0621
Fish	0.0085	0.1144	0.0601	0.0818
Fast foods	0.0646	0.0804	0.5491	0.0261
Salty snacks	0.0495	0.0854	0.6586	−0.0192
Chocolate confectionary	0.1712	0.0596	0.4463	−0.0332
Other confectionary	0.0078	0.1156	0.4102	0.0359

* non-parametric Spearman test.

**Table 6:** Analysis of the difference of dietary intake between male adolescent participants of the study declaring no acne-related quality of life problems and any acne-related quality of life problems within any question of the Acne Quality of Life (AQoL) scale.

	No Acne-Related Quality of Life Problems (*n* = 329)		Any Acne-Related Quality of Life Problems (*n* = 200)		*p* **
	Mean ± SD	Median (Min–Max)	Mean ± SD	Median (Min–Max)	
Vegetables	6.76 ± 7.51	4.5 * (0–40)	5.87 ± 8.25	4 * (0–70)	0.0563
Fruit	6.00 ± 6.71	4 * (0–40)	5.95 ± 7.30	4 * (0–45)	0.9044
Water	25.78 ± 29.02	12 * (0–255)	24.92 ± 25.44	15 * (0–140)	0.9366
Milk	3.94 ± 5.56	2 * (0–57)	4.34 ± 7.15	2 * (0–60)	0.5720
Other dairy beverages	2.33 ± 2.68	1 * (0–21)	2.62 ± 3.18	2 * (0–24)	0.0063
White bread	12.35 ± 12.55	8 * (0–65)	14.16 ± 15.01	8 * (0–70)	<0.0001
Wholegrain bread	4.76 ± 7.11	2 * (0–42)	4.56 ± 7.53	2 * (0–70)	0.0084
Other white cereal products	4.10 ± 6.27	3 * (0–86)	4.35 ± 4.51	3 * (0–40)	<0.0001
Other wholegrain cereal products	2.17 ± 2.96	1 * (0–21)	2.50 ± 3.58	2 * (0–30)	0.0694
Fish	1.03 ± 1.35	1 * (0–10)	1.42 ± 2.15	1 * (0–20)	0.7490
Fast foods	1.52 ± 1.65	1 * (0–10)	1.99 ± 3.24	1 * (0–35)	0.0006
Salty snacks	2.24 ± 2.19	2 * (0–14)	2.64 ± 2.47	2 * (0–13)	<0.0001
Chocolate confectionary	2.53 ± 3.27	2 * (0–40)	2.95 ± 3.69	2 * (0–32)	<0.0001
Other confectionary	2.33 ± 2.76	2 * (0–25)	2.92 ± 3.70	2 * (0–40)	<0.0001

* non-parametric distribution (Shapiro–Wilk test, *p* ≤ 0.05); ** non-parametric Mann–Whitney *U* test.

**Table 7:** Analysis of the difference of dietary intake between male adolescent participants of the study declaring no acne-related quality of life problems and any acne-related quality of life problems within any question of the Acne Disability Questionnaire (ADQ).

	No Acne-Related Problems (*n* = 129)		Any Acne-Related Problems (*n* = 400)		*p* **
	Mean ± SD	Median (Min–Max)	Mean ± SD	Median (Min–Max)	
Vegetables	5.62 ± 6.14	4 * (0–40)	6.68 ± 8.26	4 * (0–70)	0.1957
Fruit	5.21 ± 5.62	3 * (0–30)	6.23 ± 7.29	4 * (0–45)	0.1319
Water	26.46 ± 33.25	14 * (0–255)	25.13 ± 25.70	14 * (0–160)	0.6413
Milk	4.27 ± 6.83	2 * (0–57)	4.04 ± 6.00	2 * (0–60)	0.7623
Other dairy beverages	2.22 ± 2.15	2 * (0–10)	2.51 ± 3.08	2 * (0–24)	0.9640
White bread	12.89 ± 13.64	8 * (0–65)	13.08 ± 13.53	8 * (0–70)	0.8528
Wholegrain bread	4.34 ± 7.03	2 * (0–42)	4.79 ± 7.34	2 * (0–70)	0.1261
Other white cereal products	3.62 ± 4.59	2 * (0–40)	4.38 ± 5.97	3 * (0–86)	0.0548
Other wholegrain cereal products	2.09 ± 2.89	1 * (0–20)	2.36 ± 3.31	1 * (0–30)	0.6901
Fish	1.06 ± 1.36	1 * (0–7)	1.22 ± 1.80	1 * (0–20)	0.3200
Fast foods	1.75 ± 1.86	1 * (0–10)	1.68 ± 2.53	1 * (0–35)	0.2723
Salty snacks	2.60 ± 2.30	2 * (0–10)	2.32 ± 2.30	2 * (0–14)	0.1319
Chocolate confectionary	2.86 ± 4.11	2 * (0–40)	2.63 ± 3.19	2 * (0–32)	0.4732
Other confectionary	2.62 ± 3.39	2 * (0–25)	2.53 ± 3.08	2 * (0–40)	0.9090

* non-parametric distribution (Shapiro–Wilk test, *p* ≤ 0.05); ** non-parametric Mann–Whitney *U* test.

## Data Availability

Not applicable.

## References

[1] Sutaria A.H., Masood S., Schlessinger J. (2022). Acne Vulgaris. StatPearls.

[2] Hay R.J., Johns N.E., Williams H.C., Bolliger I.W., Dellavalle R.P., Margolis D.J., Marks R., Naldi L., Weinstock M.A., Wulf S.K. (2014). The global burden of skin disease in 2010: An analysis of the prevalence and impact of skin conditions. J. Investig. Dermatol..

[3] Lynn D.D., Umari T., Dunnick C.A., Dellavalle R.P. (2016). The epidemiology of acne vulgaris in late adolescence. Adolesc. Health Med. Ther..

[4] Szepietowska M., Dąbrowska A., Nowak B., Skinderowicz K., Wilczyński B., Krajewski P.K., Jankowska-Konsur A. (2022). Prevalence and quality of life of facial acne: Across-sectional study in high school students in Poland. Adv. Dermatol. Allergol..

[5] Hazarika N., Archana M. (2016). The Psychosocial Impact of Acne Vulgaris. Indian J. Dermatol..

[6] Kurokawa I., Nakase K. (2020). Recent advances in understanding and managing acne. F1000Research.

[7] Baldwin H., Tan J. (2021). Effects of Diet on Acne and Its Response to Treatment. Am. J. Clin. Dermatol..

[8] Dall’Oglio F., Nasca M.R., Fiorentini F., Micali G. (2021). Diet and acne: Review of the evidence from 2009 to 2020. Int. J. Dermatol..

[9] Podgórska A., Puścion-Jakubik A., Markiewicz-Żukowska R., Gromkowska-Kępka K.J., Socha K. (2021). Acne Vulgaris and Intake of Selected Dietary Nutrients—A Summary of Information. Healthcare.

[10] Meixiong J., Ricco C., Vasavda C., Ho B.K. (2022). Diet and acne: A systematic review. JAAD Int..

[11] Morze J., Przybylowicz K.E., Danielewicz A., Obara-Golebiowska M. (2017). Diet in Acne Vulgaris: Open or Solved Problem?. Iran J. Public Health.

[12] The Central Statistical Office in Poland. December 2019. https://bdl.stat.gov.pl/BDL/dane/podgrup/temat.

[13] Skolmowska D., Głąbska D., Guzek D. (2021). Differences in Adolescents’ Food Habits Checklist (AFHC) Scores before and during Pandemic in a Population-Based Sample: Polish Adolescents’ COVID-19 Experience (PLACE-19) Study. Nutrients.

[14] The Central Statistical Office in Poland. July 2021. https://stat.gov.pl/obszary-tematyczne/roczniki-statystyczne/roczniki-statystyczne/rocznik-demograficzny-2021,3,15.html.

[15] Polish Ministry of National Education. https://rspo.men.gov.pl/.

[16] Gupta M.A., Johnson A.J., Gupta A.K. (1998). The Development of an Acne Quality of Life Scale: Reliability, Validity, and Relation to Subjective Acne Severity in Mild to Moderate Acne Vulgaris. Acta Derm. Venereol..

[17] Motley R.J., Finlay A.Y. (1992). Practical Use of a Disability Index in the Routine Management of Acne. Clin. Exp. Dermatol..

[18] Abdelrazik Y.T., Ali F.M., Salek M.S., Finlay A.Y. (2021). Clinical experience and psychometric properties of the Cardiff Acne Disability Index (CADI). Br. J. Dermatol..

[19] Głąbska D., Guzek D., Ślązak J., Włodarek D. (2017). Assessing the Validity and Reproducibility of an Iron Dietary Intake Questionnaire Conducted in a Group of Young Polish Women. Nutrients.

[20] Głąbska D., Malowaniec E., Guzek D. (2017). Validity and Reproducibility of the Iodine Dietary Intake Questionnaire Assessment Conducted for Young Polish Women. Int. J. Environ. Res. Public Health.

[21] Głąbska D., Wojtas M., Guzek D. (2020). Development and validation of the semi-quantitative brief food frequency questionnaire to assess the magnesium intake in young women. Nutr. Diet..

[22] World Health Organization (WHO) Process of Translation and Adaptation of Instruments. https://www.coursehero.com/file/30372721/WHO-Process-of-translation-and-adaptation-of-instrumentspdf/.

[23] Smith H., Layton A.M., Thiboutot D., Smith A., Whitehouse H., Ghumra W., Verma M., Tan J., Jones G., Gilliland K. (2021). Identifying the Impacts of Acne and the Use of Questionnaires to Detect These Impacts: A Systematic Literature Review. Am. J. Clin. Dermatol..

[24] Nivette A., Sutherland A., Eisner M., Murray J. (2019). Sex differences in adolescent physical aggression: Evidence from sixty-three low-and middle-income countries. Aggress. Behav..

[25] Whitehead R.D., Cosma A., Cecil J., Currie C., Currie D., Neville F., Inchley J. (2018). Trends in the perceived body size of adolescent males and females in Scotland, 1990-2014: Changing associations with mental well-being. Int. J. Public Health.

[26] El Darouti M.A., Zeid O.A., Abdel Halim D.M., Hegazy R.A., Kadry D., Shehab D.I., Abdelhaliem H.S., Saleh M.A. (2016). Salty and spicy food; are they involved in the pathogenesis of acne vulgaris? A case controlled study. J. Cosmet. Dermatol..

[27] Halvorsen J.A., Dalgard F., Thoresen M., Bjertness E., Lien L. (2009). Is the association between acne and mental distress influenced by diet? Results from a cross-sectional population study among 3775 late adolescents in Oslo, Norway. BMC Public Health.

[28] Aksu A.E., Metintas S., Saracoglu Z.N., Gurel G., Sabuncu I., Arikan I., Kalyoncu C. (2012). Acne: Prevalence and relationship with dietary habits in Eskisehir, Turkey. J. Eur. Acad. Dermatol. Venereol..

[29] Alshammrie F.F., Alshammari R., Alharbi R.M., Khan F.H., Alshammari S.K. (2020). Epidemiology of Acne Vulgaris and Its Association With Lifestyle Among Adolescents and Young Adults in Hail, Kingdom of Saudi Arabia: A Community-Based Study. Cureus.

[30] Kirabo A. (2017). A new paradigm of sodium regulation in inflammation and hypertension. Am. J. Physiol. Regul. Integr. Comp. Physiol..

[31] Makrantonaki E., Ganceviciene R., Zouboulis C. (2011). An update on the role of the sebaceous gland in the pathogenesis of acne. Dermatoendocrinology.

[32] Burris J., Rietkerk W., Shikany J.M., Woolf K. (2017). Differences in Dietary Glycemic Load and Hormones in New York City Adults with No and Moderate/Severe Acne. J. Acad. Nutr. Diet..

[33] Smith R.N., Mann N.J., Braue A., Mäkeläinen H., Varigos G.A. (2007). A low-glycemic-load diet improves symptoms in acne vulgaris patients: A randomized controlled trial. Am. J. Clin. Nutr..

[34] Penso L., Touvier M., Deschasaux M., Szabo de Edelenyi F., Hercberg S., Ezzedine K., Sbidian E. (2020). Association Between Adult Acne and Dietary Behaviors: Findings From the NutriNet-Santé Prospective Cohort Study. JAMA Dermatol..

[35] Nguyen Q.G., Markus R., Katta R. (2016). Diet and acne: An exploratory survey study of patient beliefs. Dermatol. Pract. Concept..

[36] Burris J., Shikany J.M., Rietkerk W., Woolf K. (2018). A Low Glycemic Index and Glycemic Load Diet Decreases Insulin-like Growth Factor-1 among Adults with Moderate and Severe Acne: A Short-Duration, 2-Week Randomized Controlled Trial. J. Acad. Nutr. Diet..

[37] Smith R.N., Mann N.J., Braue A., Mäkeläinen H., Varigos G.A. (2007). The effect of a high-protein, low glycemic-load diet versus a conventional, high glycemic-load diet on biochemical parameters associated with acne vulgaris: A randomized, investigator-masked, controlled trial. J. Am. Acad. Dermatol..

[38] Kucharska A., Szmurło A., Sińska B. (2016). Significance of diet in treated and untreated acne vulgaris. Postepy Dermatol. Alergol..

[39] Skolmowska D., Głąbska D., Guzek D. (2020). Hand Hygiene Behaviors in a Representative Sample of Polish Adolescents in Regions Stratified by COVID-19 Morbidity and by Confounding Variables (PLACE-19 Study): Is There Any Association?. Pathogens.

[40] Guzek D., Skolmowska D., Głąbska D. (2020). Analysis of Gender-Dependent Personal Protective Behaviors in a National Sample: Polish Adolescents’ COVID-19 Experience (PLACE-19) Study. Int. J. Environ. Res. Public Health.

[41] Cinelli E., Fabbrocini G., Fattore D., Marasca C., Damiani G., Annunziata M.C. (2020). Safe distance, safe patients! Therapeutic management of oncological patients affected by cutaneous and mucosal adverse events during the COVID-19 pandemic: An Italian experience. Support. Care Cancer.

[42] Damiani G., Gironi L.C., Grada A., Kridin K., Finelli R., Buja A., Bragazzi N.L., Pigatto P.D.M., Savoia P. (2021). COVID-19 related masks increase severity of both acne (maskne) and rosacea (mask rosacea): Multi-center, real-life, telemedical, and observational prospective study. Dermatol. Ther..

[43] Głąbska D., Skolmowska D., Guzek D. (2021). Food Preferences and Food Choice Determinants in a Polish Adolescents’ COVID-19 Experience (PLACE-19) Study. Nutrients.

[44] Skolmowska D., Głąbska D., Guzek D. (2022). Body Mass and Emotional Eating: Emotional Eater Questionnaire (EEQ) in the Polish Adolescents’ COVID-19 Experience (PLACE-19) Study. Nutrients.

[45] Zari S., Alrahmani D. (2017). The association between stress and acne among female medical students in Jeddah, Saudi Arabia. Clin. Cosmet. Investig. Dermatol..

